# Fibrolysin in Obesity

**Published:** 1910-06-18

**Authors:** 


### Medicine.

### FIBROLYSIN IN OBESITY.

Fibrolysin has been employed for a great many different conditions, and that it has, or may have, some special influence upon certain kinds of metabolism is indicated by the way in which it has succeeded in causing resolution of scar tissue, at any rate in some cases. The difficulty is that the patient cannot take the remedy for himself, for it has to be injected hypodermically, and therefore requires very aseptic precautions in its administration. Naturally, moreover, its injection produces some amount of pain for the time being. In addition to its effects upon the resolution of the fibrous tissue it has been found to cause the adsorption and disappearance of an excess of subcutaneous fat in certain cases of obesity. This fact was accidentally discovered when the remedy was being employed in fat subjects for the cure of various surgical conditions.

Two cases illustrative of its effects upon obesity were recorded by Reidel as follows: The first patient was a married woman between forty and fifty years of age, who was so stout that she was unable to look after her house and was indeed helpless owing to her great bulk. An injury caused stiffness of her right shoulder, fibrolysin was tried for the cure of this, and 2.3 cubic centimetres were injected between the shoulder blades. Without becoming at all out of health the patient began to get less stout, so the injections were continued more or less irregularly on alternate days for over six months. During this time, without any alteration in diet and without any other treatment being adopted, the patient lost close upon two stones in weight, and two years later this lost weight had not been regained. Simultaneously the patient became much more active both mentally and physically, there being no unpleasant or untoward effects.

The second case was a servant girl aged seventeen, who was suffering from the vomiting of pregnancy. She was extremely obese and, for the relief of this, injections of fibrolysin were given in doses varying from 2.3 to 4.6 cubic centimetres as in the first case. Four pounds were lost in the first week and subsequently two pounds a week. With the advance of pregnancy the injections were discontinued, though up to the time that they were stopped the progressive benefit to the obesity was obvious.

